# Environmental Effects on Allergen Levels in Commercially Grown Non-Genetically Modified Soybeans: Assessing Variation Across North America

**DOI:** 10.3389/fpls.2012.00196

**Published:** 2012-08-27

**Authors:** Severin E. Stevenson, Carlotta A. Woods, Bonnie Hong, Xiaoxiao Kong, Jay J. Thelen, Gregory S. Ladics

**Affiliations:** ^1^Interdisciplinary Plant Group, Department of Biochemistry, Christopher S. Bond Life Science Center, University of MissouriColumbia, MO, USA; ^2^Pioneer Hi-Bred InternationalAnkeny, IA, USA; ^3^DuPont Agricultural Biotechnology, Pioneer Hi-BredWilmington, DE, USA

**Keywords:** soybean, GMO, allergens, MRM, LC-MS/MS, glycinin

## Abstract

Soybean (*Glycine* *max*) is a hugely valuable soft commodity that generates tens of billions of dollars annually. This value is due in part to the balanced composition of the seed which is roughly 1:2:2 oil, starch, and protein by weight. In turn, the seeds have many uses with various derivatives appearing broadly in processed food products. As is true with many edible seeds, soybeans contain proteins that are anti-nutritional factors and allergens. Soybean, along with milk, eggs, fish, crustacean shellfish, tree nuts, peanuts, and wheat, elicit a majority of food allergy reactions in the United States. Soybean seed composition can be affected by breeding, and environmental conditions (e.g., temperature, moisture, insect/pathogen load, and/or soil nutrient levels). The objective of this study was to evaluate the influence of genotype and environment on allergen and anti-nutritional proteins in soybean. To address genetic and environmental effects, four varieties of non-GM soybeans were grown in six geographically distinct regions of North America (Georgia, Iowa, Kansas, Nebraska, Ontario, and Pennsylvania). Absolute quantification of proteins by mass spectrometry can be achieved with a technique called multiple reaction monitoring (MRM), during which signals from an endogenous protein are compared to those from a synthetic heavy-labeled internal standard. Using MRM, eight allergens were absolutely quantified for each variety in each environment. Statistical analyses show that for most allergens, the effects of environment far outweigh the differences between varieties brought about by breeding.

## Introduction

Soybeans accumulate protein and oil during development, making the mature soybean seed a valuable commodity. For 2008/09, the farm value of soybeans in the United States neared $30 billion (http://www.ers.usda.gov/Briefing/SoybeansOilCrops/), exceeded in value only by corn. The value of soybean meal as both a food and feed source is dependent on the character of its storage protein reserves. In soybean, the most abundant storage proteins are members of the cupin superfamily; the legumins (glycinins), and vicilins (β-conglycinins; Derbyshire et al., 1976; Kinney et al., 2001; Takahashi et al., 2003; Radauer and Breiteneder, 2007), which account for approximately 70% of total seed protein. Together with cotton and maize, soybeans represent the majority of the genetically modified (GM) products developed to date. Most of the soybeans grown in the United States are GM varieties. In 2011, about 94% of all soybeans cultivated for the commercial market in the United States were GM (USDA National Agricultural Statistics Service (NASS), 2011). Soybean is considered one of the “big eight” allergenic foods in the United States. Soybean allergens are grouped into five categories: seed storage proteins Gly m 5 (β-conglycinin) and Gly m 6 (glycinin); Gly m TI (Kunitz trypsin inhibitor), Gly m Bd 28K, and Gly m Bd 30K (P34; FARRP version 10 allergen database, 2010: Ogawa et al., 2000; L’Hocine and Boye, 2007). One of the concerns involving GM soybeans, particularly in the European Union, is the potential increase in the levels of such endogenous allergens compared to those obtained with traditional breeding methods and subsequent enhancement in their sensitization or elicitation capacity. Data, however, are lacking in regard to the natural variability of endogenous allergen levels in non-GM soybean (Doerrer et al., 2010). Variability in protein expression levels may result from genetic differences (Fehr et al., 2003), environmental differences (Murphy and Resurreccion, 1984; Maestri et al., 1998), nutrient stress (Gayler and Sykes, 1985; Paek et al., 1997), use of different breeding methods (Burton, 1989; Yaklich, 2001; Krishnan et al., 2007), or interaction between genotype and the environment (Paek et al., 1997; Piper and Boote, 1999). These data are critical for describing and understanding potential differences of protein allergen levels among GM and non-GM soybean varieties (Doerrer et al., 2010).

Much of what we know about the protein component of mature soybean has come from various offline separation techniques like polyacrylamide gel electrophoresis, with or without isoelectric focusing, coupled with antibody or dye based detection methods. Two-dimensional (2-D) gels utilizing appropriate immobilized pH gradients that zoom to either acidic or basic pH ranges have been very successful at separating acidic and basic subunits of glycinin as well as the α and β subunits of β-conglycinin (Mooney et al., 2004; Hajduch et al., 2005; Natarajan et al., 2006; Danchenko et al., 2009). Achieving separation allows for quantification of the various spots using densitometry methods. Ultimately, these gel methods rely on mass spectrometry to distinguish the highly related proteins present in the various spots, which in the case of seed storage proteins exceed 100 (Agrawal et al., 2008). The multiplicity of 2-D spots that must each be accounted for in a quantitative study coincides with the biggest problem of 2-D gel-based quantification – low throughput (Rabilloud et al., 2010).

Quantification of proteins by mass spectrometry can be achieved using a technique called multiple reaction monitoring (MRM), during which signals from the endogenous protein are compared to those from a synthetic heavy-labeled internal standard (Kirkpatrick et al., 2005; Brun et al., 2009; Houston et al., 2010; Stevenson et al., 2010). More specifically, MRM analyses monitor peptides from proteins of interest, which are specific products of proteolysis often generated using the enzyme trypsin. The internal standards are synthetic peptides identical to the endogenous peptides of interest that have been labeled by the addition of a heavy isotope-containing amino acid, thereby changing its mass but nothing else. Because MRM is a form of tandem mass spectrometry (MS/MS), peptides (precursors) are fragmented during the analysis. Fragmentation is used to verify the sequence of the specific peptide of interest, which provides an additional level of specificity to the analysis. This technology is aided greatly by genome and RNA sequencing efforts that provide the sequences for proteins of interest, which allows for rapid MRM method development. Using this technology, proteins that differ in sequence by a single non-isobaric amino acid are theoretically discernable. In addition, MRM analysis using nanospray ionization is highly reproducible with replicate analyses, having less than 15% variation on average including analyses at the limits of instrument detection (Addona et al., 2009).

**Figure 1:**
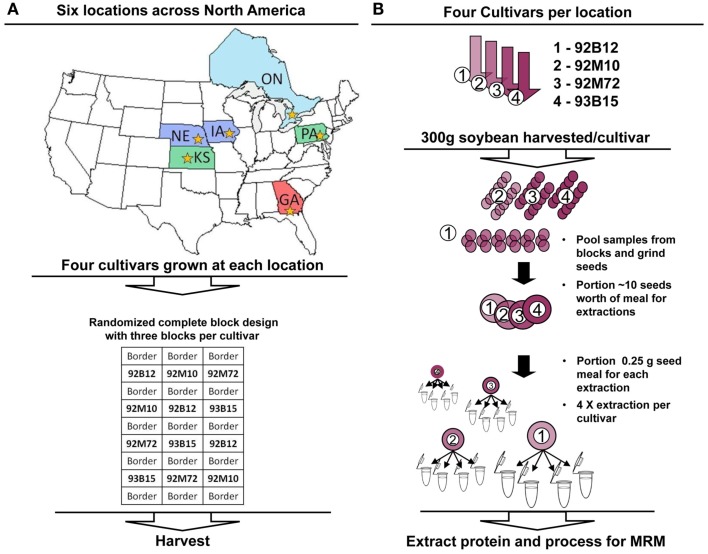
**Experimental design and processing workflows**. **(A)** Each cultivar (92B12, 92M10, 92M72, and 93B15) was grown in each location (NE, IA, PA, ONT, KS, and GA) in triplicate using a randomized complete block design for the plots. Different states/provinces in the map are colored to distinguish the three major climate zones represented within the chosen growth regions. Stars within each state/province indicate the location where the soybean cultivation occurred. **(B)** Three hundred grams of soybean was harvested from each block (each replicate). The replicate material was pooled and ground, with 10 seeds worth of meal portioned for analysis. Protein was extracted in quadruplicate from each, and analyzed using multiple reaction monitoring with AQUA™ peptide internal standards. Absolute quantitative values for soybean allergens and anti-nutritional factors were calculated and compared for variance between cultivars and environments (locations).

Recently, to begin to understand the natural variation of the allergenic protein levels in soybean, Houston et al. (2010) utilized tandem mass spectrometry to characterize the natural variation of 10 allergens (representing all five categories of soybean allergens identified to date) in 20 commercially available non-GM soybean varieties. The authors reported that the absolute quantities [absolute per unit protein (μg allergen/mg protein)] of the studied allergens extended over a 10-fold range. The objective of the current study is to further evaluate the influence of genotypes and environments. Four non-GM commercially available varieties of soybeans were each grown at six different locations (Georgia, Iowa, Kansas, Nebraska, Ontario, and Pennsylvania, Figure 1). Soybean allergens were measured similarly to Houston et al. (2010) and absolute quantities of soybean allergens reported. Principal Component Analysis (PCA) was conducted to explore the patterns of multivariate data and a biplot (Gabriel, 1971) was utilized to provide results graphically. Genotypic variation and environmental variation were quantified relative to the overall mean and relative to residual variation using Analysis of Variance (ANOVA)-based *F*-test. The data suggest that over a broad geographical region, the environment plays a larger role than genotype in determining allergen/anti-nutrient protein levels.

## Materials and Methods

Unless stated otherwise, all reagents were purchased through Research Products International, Mount Prospect, IL, USA.

### Soybean cultivation, collection, and processing

Four commercially available non-GM soybean varieties (92B12, 92M10, 92M72, and 93B15 were utilized for this study. A similarity matrix using all the markers available that are in common between the varieties was conducted and it was found that the lines have anywhere from 62 to 71% similarity. The study design included six sites located in the commercial soybean-growing regions of North America: Quitman, GA, USA; Richland, IA, USA; Larned, KS, USA; York, NE, USA; Branchton, ON, Canada; and Hereford, PA, USA. The field phase of this study was conducted during the 2007 growing season. The mean temperature during the day for each site was as follows: GA (90.5°F); IA (81.5°F); KS (86.5°F); NE (80.3°F); ON (77.5°F); PA (86.8°F). The mean temperature during the night for each site was as follows: GA (68.2°F); IA (60.5°F); KS (59.2°F); NE (58.7°F); ON (53.5°F); PA (58.2°F). Average rainfall for each site is as follows: GA (4.98″); IA (4.79″); KS (2.23″); NE (3.45″); ON (1.99″); PA (2.87″). A randomized complete block design containing three blocks, with each block containing the 92B12, 92M10, 92M72, and 93B15 commercial soybean varieties, was utilized at each site. Each soybean variety was planted in a two-row plot, which was bordered on either side by one row of non-GM commercial soybean variety of similar relative maturity. Sites were surrounded by at least 10 feet of bare ground buffer area. All plants grew well at all locations and harvesting was conducted at typical maturity/dryness levels. Typical would refer to 95% of pods on the plant are mature and seed weight adjusted to 13% seed water content. Seed samples for each cultivar (300 g from each block) were harvested and pooled. There was no morphology/physiology differences among the seeds collected from the different locations. The pools were ground and a portion removed for each cultivar. Samples were shipped at ambient temperatures to the University of Missouri for analysis. Samples were stored at room temperature in the dark until extracted.

### Protein isolation and purification

Protein was isolated in quadruplicate from 0.25 g of soybean meal using the extraction method described in (Stevenson et al., 2009). Briefly, soybean meal was suspended in 2.5 mL of Tris-buffered phenol (pH 8.0; Fisher Scientific, Inc., Pittsburgh, PA, USA) and 2.5 mL extraction buffer [0.9 M sucrose, 0.1 M Tris-HCl pH 8.0, 10 mM EDTA, 0.4% (v/v) 2-mercaptoethanol]. The mixture was homogenized using a T25 Basic S1 Disperser (IKA Works, Inc., Wilmington, NC, USA) on speed 4 with the S25N-10 tool. The emulsion was inverted at 4°C for 2 h and then centrifuged at 5000 × *g* and 4°C for 20 min. Phenol was removed and a single back extraction was performed in the same manner. Phenol fractions were combined and protein precipitated using five volumes of 95% (v/v) methanol with 0.1 M ammonium acetate and incubation at −20°C overnight. Precipitated protein was pelleted by centrifugation at 5000 × *g* and 4°C for 5 min, and washed by re-suspending in 10 mL fresh methanol-ammonium acetate solution three times, 80% (v/v) acetone two times, and 70% (v/v) ethanol once, with 20 min incubations at −20°C before pelleting. Protein was stored in 80% (v/v) acetone at −20°C.

### Preparation for mass spectrometry

#### Total protein quantification by Bradford dye-binding

Twenty-five microliters of precipitated protein was collected by centrifugation at 16,000 × *g* at 4°C for 1 min. Pellets were dissolved in 300 μL of urea buffer (50 mM Tris-HCl, pH 8.0, and 8 M urea) by pipetting and vortexing, and finally centrifuged at 16,000 × *g* at 4°C for 10 min to pool and pellet insoluble material. Supernatants were collected, split into two equal portions and snap frozen in liquid nitrogen and stored at −80°C. Dissolved protein samples were thawed at 4°C, and vortexed to make homogenous. Samples were quantified in quadruplicate using 3 μL of undiluted sample per replicate. Quantification was accomplished using the Coomassie dye-binding assay at 1× (Bio-Rad, Hercules, CA, USA) using the 96-well plate format employing an in-house-prepared soy protein (Williams 82) standard at 2 mg/mL in urea buffer. A five point standard curve of 0 (3 μL of urea buffer), 1, 2, 4, and 8 mg/well was created using a twofold dilution series made with urea buffer. The standard curve used 4 μL/well, and was done in triplicate. Final protein concentration was the average of the protein concentrations obtained from the four replicate assays. After the concentrations were determined, the reconstitution step was repeated using back-calculated values for volume (using between 200 and 800 μL/sample) in order to reconstitute each sample to a concentration of 1–2 mg/mL. Protein quantification was repeated in the same manner, using one of the frozen aliquots, to verify the final concentrations were between 1 and 2 mg/mL.

#### In-solution digestion

The remaining aliquot of frozen protein was thawed at 4°C, vortexed to make homogenous and 10 μg of each sample was portioned into 1.5 mL polypropylene tubes. For each, the volume was brought to 10.0 μL using 8 M urea buffer. Disulfide bonds were reduced with 15.0 μL of reduction solution (16.7 mM dithiothreitol (DTT), 6.7 ng/μL BSA in 50 mM ammonium bicarbonate) for 1 h at room temperature. Reduced cysteines were carboxyamidomethylated with 5.0 μL alkylation solution (300 mM iodoacetamide in 50 mM ammonium bicarbonate) at room temperature in the dark for 1 h. Urea was diluted to 0.67 M and iodoacetamide was neutralized with equimolar DTT by adding 90 μL of neutralization solution (16.7 mM DTT in 50 mM ammonium bicarbonate) and incubating at room temperature for 15 min. Samples were chilled to 4°C and 10.0 μL of cold trypsin solution (20 ng/μL sequencing grade – modified, Promega, Madison, WI, USA) in 50 mM ammonium bicarbonate) added. Digestion was allowed to proceed for 16 h at 37°C. AQUA™ peptides (Sigma Life Science, The Woodlands, TX, USA) in 50% acetonitrile (ACN), 1.0% (v/v) formic acid were added to the completed digests, which were frozen and evaporated to dryness using a CentriVap before being stored at −80°C.

#### AQUA™ peptide preparation and storage

AQUA™ peptides were synthesized by Sigma-Aldrich Co. LLC., to greater than 95% purity, and were portioned in 0.5 nmol aliquots and stored lyophilized at −20°C until use. AQUA™ peptides were dissolved to 2.5 pmol/μL using 50% (v/v) ACN (Sigma-Aldrich, Saint Louis, MO, USA), 1.0% (v/v) formic acid (Acros Organics, New Jersey, USA) with 5 × 10 s of vortexing max speed. Portions of 100 pmol were made by aliquoting 40 μL to separate tubes and storing at −80°C after centrifugal evaporation. Prior to use, peptide aliquots were dissolved in 50% (v/v) ACN, 1.0% (v/v) formic acid in an appropriate volume so that when mixed together, the final AQUA™ concentration is 50 fmol/μL (except for glycinin G1, which was at 100 fmol/μL). AQUA™ peptides at these concentrations were added to completed digests at 2 μL/μg of digested material.

### Absolute quantification with MRM

#### Sample loading and mass analysis

All sample digests were dissolved to 200 ng/μL with 5% (v/v) ACN, 0.1% (v/v) formic acid with vortexing (3 × 10 s max speed). Insoluble debris was pelleted with centrifugation at 21,000 × *g* for 2 min at room temperature. Clearspun samples were immediately transferred to a 96-well plate, covered with film and placed in the 8°C autosampler tray. All mass spectrometry was performed using a TSQ Vantage Extended Mass Range triple quadrupole mass spectrometer (Thermo Fisher Scientific, San Jose, CA, USA). Ion optics were tuned using Angiotensin at 500 fmol/μL [50% (v/v) methanol, 1.0% (v/v) formic acid], and capillary temperature step optimized for peptide desolvation under normal LC workflows with purified BSA peptides (Michrom Bioresources, Inc., Auburn, CA, USA) using the gradient described below. Sample handling and LC separations for LC-SRM analyses were all performed using an Eksigent nanoLC ultra 1D plus [0.1% (v/v) formic acid in MilliQ water (18.2 MΩ) as solvent A, and 99.9% (v/v) ACN, 0.1% (v/v) formic acid as solvent B]. Peptides were trapped and washed on a C_8_ Cap Trap (Michrom Bioresources, Inc., Auburn, CA, USA) using solvent A at 5 μL/min for 2 min. Peptide chromatography used a 25 min non-linear gradient from 2 to 60% over 12.5 min, a washing step, and a 9 min equilibration step at 2% solvent B all using a flow rate of 500 nL/min. AQUA™ peptides used were designed and described in Houston et al. (2010). All are listed in Table A1 in Appendix. Precursor and product ions, ion types, and charge states are also listed in Table A2 in Appendix. The development of an optimal LC-MRM analysis, including most abundant precursor and product ion selection and collision energy optimization, was aided by iterative analysis of soybean tryptic digests using Pinpoint 1.1 (Thermo Fisher Scientific, 2011). Peptide sequences were entered into Pinpoint manually and the recommended workflows for precursor/product ion selection were followed. When necessary, precursor and product ions were chosen manually by direct infusion of peptides at 500 fmol/μL in 50% (v/v) ACN, 1.0% (v/v) formic acid using product ion scans from the most intense precursor. At least five transitions were chosen for each peptide. Cycle times and chromatography were optimized to ensure 10 measurements for each transition across the chromatographic peak profile.

#### Data analysis

Selected Reaction Monitoring results were provided by LCQuan 2.6 (Thermo Fisher Scientific, 2009). Peak detection and integration were performed using the ICIS algorithm with five points of smoothing, and a baseline window of 20. All other values were left as default. Results were exported from LCQuan as tab delimited text files which were imported into MS-Excel (2007) for calculations. Response ratios (unlabeled peptide area/labeled peptide area) were multiplied by the mole amount of each AQUA™ present per microgram of sample to obtain endogenous mole quantities. Endogenous mole quantities were multiplied by the molecular weight of the protein (Table A1 in Appendix), and grams per microgram was converted to microgram protein per milligram of digest by dimensional analysis.

#### Determining linear ranges

Linear ranges of detection for all unlabeled peptides of interest were determined with a twofold dilution series of soybean total protein digest using 0.5 M urea buffer (similar to final digest buffer) and AQUA™ internal standards at 100 fmol on column. Injection amounts for the dilution series were from 2 μg down to 125 ng on column. Using the peptide area ratios (unlabeled/labeled) attained from this initial standard curve, linear ranges were determined for each peptide by manually inspecting the response ratio changes between dilutions, using a twofold change in area ratio as an indicator of a perfect response between two dilutions. Slight deviations from this ideality were accounted for by ensuring the internal standard peptide was within 10-fold of the endogenous peptide signal (response of 10–0.1) for all peptides. Final quantitative analyses were performed with labeled standards at these concentrations. In most cases 100 fmol/μg of soy digest was appropriate. AQUA™ internal standard peptides were also analyzed for their linearity of response using a twofold dilution series from 2 pmol/injection down to 0.061 fmol/injection. This dilution was performed using total soy protein digest material at 200 ng/μL (typical endogenous peptide concentrations). All peptides, endogenous and AQUA™, were used within their linear ranges.

### Statistical methods

#### Technical variation to assess the reproducibility of MRM replicate analyses

Technical variation was quantified by coefficient of variation (%CV). The %CV for a single variable (i.e., soybean allergen) aims to describe the measurement dispersion of the variable in a way that does not depend on the variable’s measurement unit. The higher the %CV is, the greater the measurement dispersion is in the variable. For each soybean allergen, %CV was calculated for each cultivar in each site using the following formula:

$$\%{\text{CV}}_{ij}=\frac{s(y_{ij})}{y(y_{ij})}\times 100$$

where %CV*_ij_* is the %CV for the *i*th cultivar at the *j*th site (*i* = 1, 2, 3, 4, *j* = 1, 2, …, 6), and *s*(y*_ij_*) and $\bar{y}\left(y_{ij}\right)$ are sample standard deviation and sample mean of four replicate individual data values *y_ijk_* (*k* = 1, 2, 3, 4), respectively.

#### ANOVA analysis to assess genotypic variation and environmental variation

For a given variable, genotypic variation and environmental variation were quantified relative to residual variation using ANOVA-based *F*-test. Genotypic variation and environmental variation were also assessed using %CV, which represents normalized variation to the overall mean of the variable. ANOVA was conducted using the following statistical model:

$$y_{ijk}=\text{U}+{\text{G}}_{i}+E_{j}+e_{ijk}$$

where *y_ijk_* denotes the *k*th measurement for the *i*th cultivar at the *j*th site (*i* = 1, 2, 3, 4, *j* = 1, 2, …, 6, *k* = 1, 2, 3, 4); *U* denotes the overall mean (fixed effect); *G_i_* denotes the *i*^th^ genotype effect (fixed effect); *E_j_* denotes the *j*th environment effect (fixed effect); and *e_ijk_* denotes the residual term associated with *y_ijk_* (random error). The assumption for residual terms is *e_ijk_* ∼ MVN(0, Σ) where MVN denotes multivariate normal and Σ denotes variance-covariance matrix. The Compound Symmetry model was utilized to fit the residual variance-covariance structure for repeated measurements on the same subject (a subject was defined as a unique cultivar by site combination).

#### Principal component analysis

A PCA analysis was conducted to explore the patterns of multivariate data and a biplot (Gabriel, 1971) was utilized to provide results in graphic presentation (Figure 5). The biplot visualizes the relationships among allergens and identifies allergens that are positively or negatively associated. It is also useful to visualize the allergen profiles of samples (site and cultivar combinations).

## Results

### Technical variation to assess the reproducibility of MRM replicates

Small technical variation was observed for Glycinin G1, Glycinin G2, and KTI 1 with an average %CV less than 10 and a majority of %CV values being 5–15. Medium technical variation was observed for Beta conglycinin α subunit, Gly m Bd 28k, Glycinin G3, Glycinin G4, and KTI 3 with average %CV of less than 15 and a majority of the %CV values being 5–25.

### Statistical assessment of variation trends using ANOVA analysis

**Figure 2:**
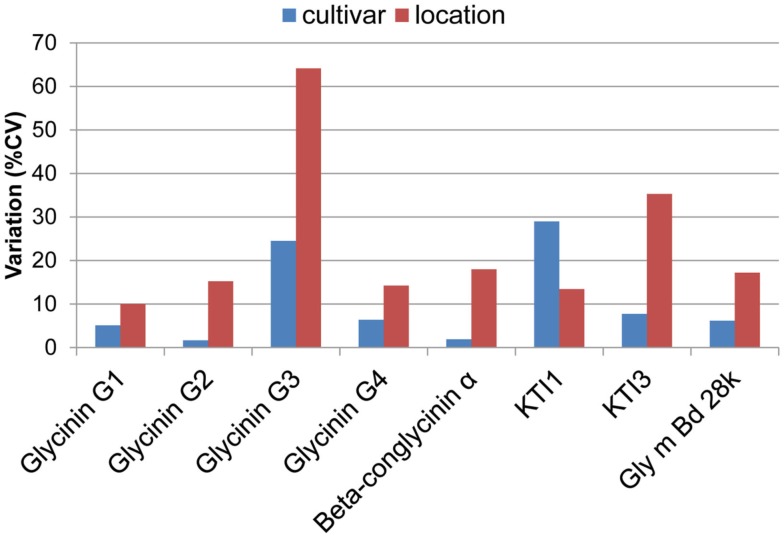
**Environmental and varietal variation**. Percent coefficients of variation (%CV, standard deviation(−mean × 100) for allergen quantities are plotted for each allergen with respect to environment and cultivar. The variation was normalized with respect to the mean. This variation was also analyzed using ANOVA, presented in Table 1.

**Figure 3:**
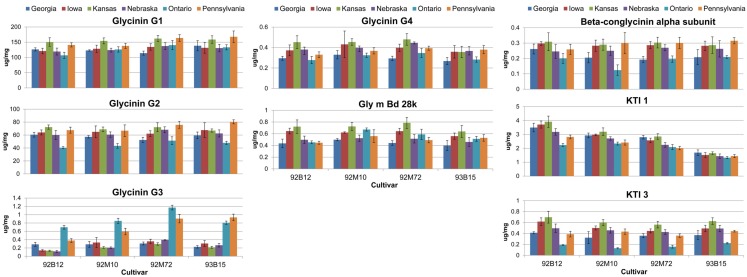
**Absolute quantities for allergens and anti-nutritional proteins**. The absolute quantities for the eight proteins of interest are presented. Data are grouped by cultivar with each separate bar representing the quantity of the protein for the indicated environment (location). Error bars are standard deviation between extraction replicates.

Genotypic and environmental variation assessed using %CV are shown in Figure 2. From this plot, the amount of variation observed was larger between locations than between cultivars, with KTI 1 being the only exception, showing over onefold greater variation from the cultivar than the location. KTI 3 showed a similar differential in variation but in an opposite direction, with the location providing over threefold greater variation than the cultivar. KTI 1 expression was influenced more by the genotype and KTI 3 was affected more by the environment based on the *F* statistics and *P* values (Table 1). KTI 1 expression was the only allergen clearly influenced by the cultivar in this study compared to the other allergens with an *F*-value of 61.57 (Table 1). Cultivar 92B12 had the highest amount of KTI 1 protein and cultivar 93B15 had the lowest (Figure 3). The expression trend of KTI 3 was similar among the cultivars. The environment had the greatest impact on KTI 3 expression in this study compared to the other allergens, with an *F*-value of 70.54. Cultivar 92B12 had higher levels of KTI 3 compared to the other three commercial cultivars (Figure 3). However, all the cultivars grown in Ontario had low levels of KTI 3 compared to the other locations. KTI 3 levels were twofold to fourfold– reduced for each cultivar grown in Ontario. These drastic differences in variation between the two variables (cultivar and location) were unique to the KTIs. Four paralogs for the seed storage protein glycinin were individually quantified based upon unique peptides, which revealed a surprising range of expression variation. The most prominently expressed paralog, Glycinin G1, showed 5% variation with respect to cultivar and 10% for location, indicating that neither variable was causing large expression changes, when considering %CV. Glycinin G2 also showed low overall variation, however, the disparity between variation from location and cultivar was large, 15 and 2% respectively. In contrast, one of the two lower abundance forms, Glycinin G3, has the largest percent variation. Location resulted in variation exceeding 60%, which was approximately twice the variation observed among cultivars.

**Table 1:** **ANOVA analysis of allergen levels**.

Variable	Effect	NumDF	DenDF	*F-*value	ProbF
Glycinin G1	Site	5	15	12.88	<0.0001
Glycinin G1	Cultivar	3	15	5.61	0.0088
Glycinin G2	Site	5	15	22.67	<0.0001
Glycinin G2	Cultivar	3	15	1.39	0.2850
Glycinin G3	Site	5	15	26.72	<0.0001
Glycinin G3	Cultivar	3	15	6.62	0.0046
Glycinin G4	Site	5	15	18.07	<0.0001
Glycinin G4	Cultivar	3	15	6.11	0.0063
Beta conglycinin alpha	Site	5	15	15.38	<0.0001
Beta conglycinin alpha	Cultivar	3	15	0.92	0.4558
KTI 1	Site	5	15	9.67	0.0003
KTI 1	Cultivar	3	15	61.57	<0.0001
KTI 3	Site	5	15	70.54	<0.0001
KTI 3	Cultivar	3	15	6.00	0.0068
Gly m Bd 28k	Site	5	15	19.07	<0.0001
Gly m Bd 28k	Cultivar	3	15	4.47	0.0197

*For a given variable, genotypic variation, and environmental variation were quantified relative to residual variation using Analysis of Variance (ANOVA)-based *F*-test*.

The levels of Glycinin G1 and G2 were affected by the environment and to a lesser extent by the cultivar (Table 1). Kansas and Pennsylvania had significantly higher levels of Glycinin G1 compared to the other locations across four cultivars (Figure 3). Conversely, Ontario had significantly lower levels of Glycinin G2 compared to the other locations across four cultivars. The level of Glycinin G3 was impacted by both the environment and the cultivar, but the environmental factor had a greater impact on the expression of this allergen as indicated by both the *F* statistics and *P* value (Table 1). Ontario and Pennsylvania had significantly higher levels of Glycinin G3 compared to the other locations across four cultivars.

The levels of Glycinin G4 were affected by the environment (Table 1); with cultivars grown in Georgia and Ontario having the lowest levels (Figure 3). The difference in cultivar also affected the expression of Glycinin G4 (Table 1). Cultivar 92B12 and 93B15 had the lowest levels of Glycinin G4 across all six locations. The expression level of Gly m Bd 28k and β-conglycinin α subunit were affected mainly by the environment it was grown in (Table 1). Iowa and Kansas had the highest levels of Gly m Bd 28k compared to the other sites across four commercial varieties (Figure 3). In contrast, cultivars grown in Georgia were consistently lower than those grown in Iowa and Kansas (Figure 3). Nevertheless, a lower level of β-conglycinin α subunit was observed in all the cultivars grown in Ontario (Figure 3).

### Total allergen content of four commercial varieties grown in six different locations

**Figure 4:**
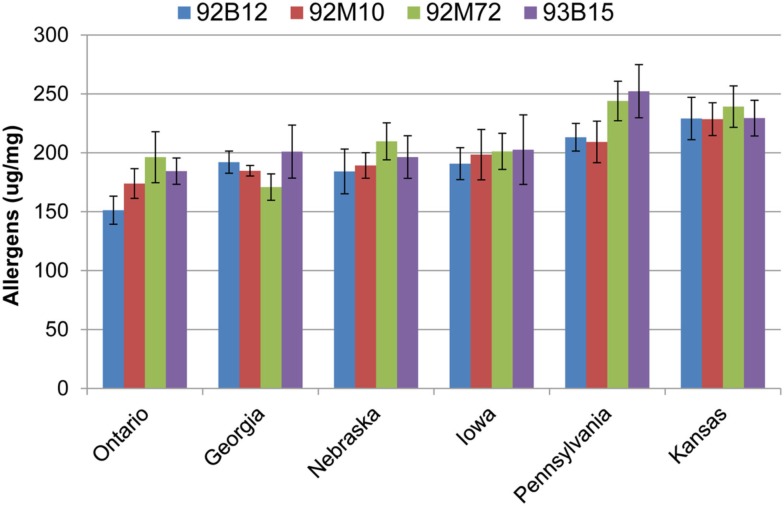
**Allergen totals by location**. Values presented are the sum totals for all eight proteins measured. Data are grouped by location, and error bars are summed standard deviations for each allergen. Groups were ordered according to their average allergen sums.

The total allergen content was similar between Ontario, Georgia, Nebraska, and Iowa (Figure 4). Likewise, the allergen content was similar between Pennsylvania, and Kansas. Cultivar 92B12 grown in Ontario had the lowest level of total allergens whereas; cultivars 92M72 and 93B15 grown in Pennsylvania had the highest levels of total allergens (Figure 4).

### Principal component analysis

**Figure 5:**
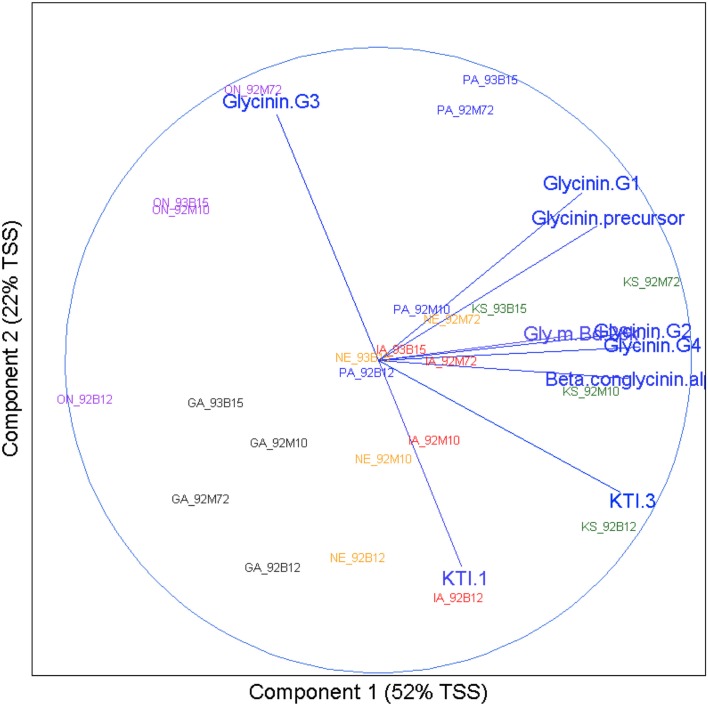
**Environment versus cultivar biplot**. Results of principal component analysis were visualized using a biplot. The *x* axis represents the direction of maximum variation through the data, which is called the first principle component direction. The *y* axis represents the direction of the next highest variation through the data, which is orthogonal to the first principle component and called the second principle component direction. Text in the body of the plot represents the samples. The name for each has two parts: location abbreviation followed by cultivar number. Correlations between allergens are represented by vectors terminating with text indicating the allergen they represent. Angles between these vectors, acute or obtuse, represent positive or negative correlation respectively.

The following can be seen from Figure 5: (1) Across the 24 samples (four cultivars at six sites), Glycinin G2, Glycinin G4, and Beta conglycinin α were positively correlated (a small acute angle), and KTI 3-1 and KTI 3-2 were positively correlated (an acute angle). Glycinin G3 was negatively correlated with Glycinin G2, KTI 1, KTI 3, Beta conglycinin α, and Glycinin G4 (obtuse angles). (2) Samples tended to be clustered together by sites. All four cultivars from the GA site were at the lower left quadrant of the biplot. All four cultivars from the ON site were at the left part of the biplot. All four cultivars from the PA site were at the upper part of the biplot. All four cultivars from the KS site were at the right part of the biplot. All four cultivars from IA and NE sites were at the lower part of the biplot and showed similar pattern by cultivar. From PCA analysis it can be concluded that variation among sites (i.e., environment) was the dominant variation for allergens.

## Discussion

### Considerations for throughput optimization

In this study, eight allergens were quantified in 24 samples with each sample measured four times. In order to perform this ambitious study we addressed the individual seed variability by taking large samplings to average this component. This was performed using standard procedure for random sampling of material from test plots. It should also be noted that subsequent quantification was performed on single representative AQUA™ peptides as explained in the Section “Materials and Methods.”

**Figure A1:**
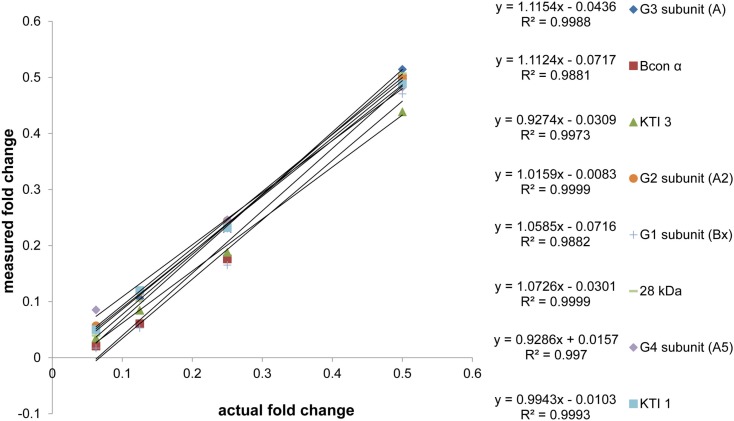
**Matrix dilution series**. The linearity of response of endogenous peptides was assessed with constant AQUA™. A twofold dilution series from 2 to 0.125 μg/injection of soybean (Williams 82) digest was analyzed. The fractional changes, from undiluted through the twofold dilution series, both measured (via response ratio) and actual, were plotted to assess the slope and correlation values. Values on the *x* and *y* axes are the fraction of undiluted sample (2 μg/injection) for each successive dilution in the series (1, 0.5, 0.25, and 0.125 μg/injection). Technical variation for the experiment was on average, 8.86% when considering coefficients of variance for triplicate injections of all peptides at all dilutions.

Once protein was extracted, processing from protein dissolution to calculations for absolute quantity took approximately 6 days, with an average percent coefficient of variance for the experiment at 10.0%. In order to achieve this rapid turnover, some assumptions had to be made. Firstly, we assumed that replicate injections could be forgone if replicate extractions were performed for each sample. The experimental design employed replicate extractions to assess variation at the protein extraction level. Assuming that the variation at the extraction level was greater than the variation at the injection level (largely controlled for with AQUA™ peptides, and previously assessed at 3–5%), we were satisfied that we were accounting for the majority of experimental variation by comparing replicate extractions without replicate injections. Secondly, we assumed that single point quantification can accurately assess peptide abundance. For our quantification experiments, protein quantities were calculated based on a single simultaneous measurement of both AQUA™ and endogenous peptide intensities. Ideally, the analysis would be repeated for each sample using a dilution strategy, where the AQUA™ peptide or the endogenous matrix is diluted and subsequent measurements are made of these samples to assess the linearity of response. To avoid this necessity, we assessed the linearity of our endogenous peptides with a twofold dilution series from 2.0 to 0.0625 μg/injection (2.0 μg maximum capacity peptide traps) of a Williams 82 soybean protein sample using a constant amount of AQUA™ peptide for all dilutions. The ratio of endogenous: AQUA™ peak areas were used to assess linearity. The scatter plot of “measured fold change” versus “actual fold change” shows slopes that on average are within 5.70 ± 3.82% of the optimal value of 1, with an average *R*^2^ value of 0.9969 ± 0.0042 (Figure A1 in Appendix). With this information in mind, we decided to use 1 μg injections allowing for at least a twofold increase, and a 16-fold decrease in any of the endogenous peptides without extrapolating past the linear range (2.0–0.0625 μg). To maximize the efficacy of our internal standards, we calibrated the amount of each AQUA™ to approximately match the amount of the endogenous analyte. This relationship was not maintained for two of the peptide analytes (Glycinin 1 and Glycinin 2) which were present in such large quantities that matching the signal would require 3 and 2 pmol respectively per injection. For these peptides, a 10:1 (endogenous: AQUA™) ratio was attained, which was still within the pre-determined linear range.

Lastly, our strategy assumed that monitoring one proteotypic peptide is sufficient to represent the whole protein. This assumption is mostly an assumption of complete protein digestion. Although digestion conditions were monitored by Coomassie SDS-PAGE to ensure all proteins were digested to peptides, this approach is not as quantitative as MRM. Ideally, if a protein is subjected to proteolysis using trypsin, the entire protein will be reduced to peptides by cleavage at all Lys and Arg residues. However, this is not necessarily the case. Studies have shown that a number of exceptions exist, which prevent cleavage, or prevent complete cleavage. For example, trypsin was perceived to not cleave at a Lys or Arg when a Pro immediately follows it (Keil, 1992). This rule has been questioned recently. A group using peptide identification data from both low and high mass accuracy proteomics experiments have shown that the prevalence of cuts at the Lys or Arg residues preceding Pro indicates they are not preferred by trypsin but are in fact possible (Rodriguez et al., 2007). The presence of a double cleavage site can also cause issues. If two Lys or Arg residues are immediately adjacent or within a few residues, there is the possibility of incomplete cleavage at any one of the residues. Furthermore, accuracy of quantification is affected by chemistry. Depending on when the internal standards are added (e.g., immediately before, versus immediately after the digestion step), chemical modifications could alter the peptide pool, potentially altering the mass of the endogenous peptide and thereby preventing its measurement during MRM. In our experience, peptide loss due to breakdown or modification is highly dependent on the peptide sequence, with certain combinations of amino acids being more prone to internal or external chemistry. For all of these reasons, using multiple peptides is likely to provide more accurate results. However, with all of the cleavage and chemistry rules considered, most allergens that we wanted to measure have only a single peptide suitable for quantification.

### Measured allergen levels

It has been shown that 50–70% of total seed protein is contributed by the storage proteins glycinin and β-conglycinin with their individual percentages varying drastically between studies (Wolf et al., 1961; Saio, 1969; Murphy and Resurreccion, 1984). In our study, cumulative allergen content was lower than expected, with totals approaching 25% (w/w). We believe this is caused by several factors, including fractional quantitation of all paralogous protein forms and alternative protease cleavage sites.

Quantitation of the β-conglycinin fraction was by far the most incomplete which is largely because peptides for β-conglycinin subunits α′ and β were not analyzed in this study, thereby vastly under-representing the β-conglycinin fraction. Secondly, the peptide used for β-conglycinin α may be under represented due to the presence of an “illegitimate” cleavage site (Keil, 1992) that may in fact be partially cleaved *in vitro* (Rodriguez et al., 2007). Because this peptide is quantifiable, it is obviously not completely cleaved, and therefore may still be useful for quantification if the AQUA™ peptides are spiked prior to digestion, assuming trypsin is capable of stoichiometrically cleaving this small synthetic peptide. Although this observation would affect the absolute levels of this protein, it should not influence relative levels as the protein would likely be equally processed *in vitro*. The glycinin fraction was the most prevalent in this study, approaching an additive total of nearly 200 μg/mg (∼20%, w/w). This value is still lower than values provided in the literature (ca. 40%), however a large portion of this discrepancy is likely due to the fact that not all glycinin forms were quantified including glycinin subunit 5 (Gly G5). A previous report on soybean allergens using the same quantitation technology reported substantially lower levels of these storage proteins (Houston et al., 2010). However, the values for that study were for peptide concentrations not proteins which required correction to intact protein molecular mass as listed in the methods. Alternative methods, such as protein ELISA, were not commercially available for the individual allergens in this study.

### Practical considerations of allergen variation

The levels of allergens have been previously reported to vary between non-GM varieties. For example, in a study of non-GM soybean varieties involving skin reactivity and *in vitro* IgE binding of 10 soybean cultivars, Codina et al. (2003) reported up to sixfold differences in IgE binding potencies, while Sten et al. (2004) reported wide variation in IgE binding to different varieties of the same species of non-GM crops. To investigate an allergen’s natural variation in expression, Xu et al. (2007) evaluated 16 soybean varieties and showed relative variation in allergens, such as Gly m 5 and Gly m Bd 30K, between wild and cultivated non-GM soybean varieties. Houston et al. (2010) quantified soybean allergens in 20 non-GM varieties and observed anywhere from statistically insignificant changes in expression for some allergens to as much as 10-fold for glycinin G3 (a Gly m 6 isoform) when comparing two different varieties.

These examples give an indication of variation in the allergen levels of various non-GM soybean brought about by breeding. Further research is needed to establish a “baseline” of allergen levels from a composite of both the genetic and even broader environmental factors. Toward this goal, we report here a comparison of genetic versus environmental variation in the expression of eight soybean allergens. While the genetic variation in this study was limited (i.e., early, edible ancestors of soybean were not studied), the environmental component was vast – spanning three agriculturally relevant climate zones (4–6) covering a large range of North America. The results indicate a greater influence of environment on allergen variation, although this was dependent upon the allergen in question, suggesting that protein function is the determining factor in a “genetic versus environmental” discussion. At this point in time, it is apparent that currently one cannot simply speculate or predict how allergens will respond to either a changing genetic background or environment.

While scientifically interesting, this does not directly address the concerns of regulatory safety. The current human health safety evaluation of GM food crops involves an evaluation of endogenous allergen levels using serum IgE screening when its’ non-GM equivalent is a commonly allergenic food (e.g., soybean; Holzhauser et al., 2008; Thomas et al., 2008). Given the natural variation in levels of endogenous allergens of available non-GM food crops due to differences in: (1) the genetics of commercial varieties (Houston et al., 2010) and (2) the interactions of those varieties with the environment (i.e., temperature, moisture, nutrients, plant pathogens, insect loads; Sancho et al., 2006a,b; Goodman et al., 2008; Doerrer et al., 2010), it is evident that we are only beginning to understand the contextual importance of natural variation in allergen assessment. It is important to note that no safety associated information is gained by performing endogenous allergen comparison studies on GM soybean if the analysis isn’t compared to non-GM soybean levels (i.e., natural variability) already in the food supply. With antibody-based assays that monitor allergen expression, it is necessary to monitor each of these non-GM reference varieties along with the GM line, simply because the assays are either relative or non-specific. The AQUA™-MRM approach employed here does not have these limitations and could conceivably allow for direct monitoring of only the GM line, once sufficient reference data have been acquired and compiled in a public database for comparative purposes.

## Conflict of Interest Statement

Severin E. Stevenson, Carlotta A. Woods, and Jay J. Thelen claim no conflicts of interest. Gregory Ladics, Bonnie Hong, and Xiaoxiao Kong are employed by Pioneer Hi-Bred, a DuPont Business that makes GM crops.

## Appendix

**Table A1:** **Allergen peptide analyte information**.

	Nomenclature		Peptide information			Protein information	
ID no.	Peptide name	Protein represented	Peptide sequence	Calculated (Da)	Observed (Th)	Glyma no.	Protein MW (g/mol)monoisotopic (M+H)1+
**Gly m 6 (GLYCININ)**							
SA2	GlyG1-2	G1 subunit (Bx)	VLIVPQNFVVAAR	1425.857604	713.4	Glyma03g32030.1	55671.6
SA4	GlyG2-2	G2 subunit (A2)	NLQGENEEEDSGAIVTVK	1931.919212	966.5	Glyma03g32020.1, Glyma03g32020.2	54356.887636, 41045.114403
SA6	GlyG3-2	G3 subunit (A)	FYLAGNQEQEFLQYQPQK	2231.076708	1116.0	Glyma19g34780.1	54207.863911
SA7	GlyG4	G4 subunit (A5)	VESEGGLIQTWNSQHPELK	2152.066872	718.0	Glyma10g04280.1	63758.458975
**Gly m 5 (BETA CONGLYCININ)**							
SA10	Bcon α	Alpha subunit	LITLAIPVNKPGR	1391.873253	464.6	Glyma20g28650.1, Glyma20g28650.2, Glyma20g28660.1	70263.437646, 63248.763394, 70263.437646
**Gly mTI (KUNITZ TRYPSIN INHIBITOR)**							
SA11	KTI 3-1	KTI 3	FIAEGHPLSLK	1211.678251	404.6	Glyma08g45530.1	14517.404983
SA13	KTI 1	KTI 1	DTVDGWFNIER	1351.627676	676.3	Glyma01g10900.1	22432.404593
**Gly m BD (P34)**							
SA14	AllGly28	28 kDa	DGPLEFFGFSTSAR	1530.7223	765.9	Glyma11g15870.1	52882.740099

*Soy peptide data – Peptide sequences, masses, and observed masses for all peptides used in absolute quantification experiments are presented. In addition, the name of the annotated Swiss-Prot entry (“Protein represented” column) that corresponds to the soybean genome sequence (“Glyma no.”, Schmutz et al., 2010; www.phytozome.org) used to design the peptide, is presented. In the case where multiple splice variants were present, the underlined information was used. The uncharged monoisotopic mass of the protein sequences represented by the Glyma numbers which were used in molar quantity calculations, are presented*.

**Table A2:** **Allergen peptide MRM information**.

	Precursor (Th)	Precursor charge	Product (Th)	Product charge	Ion type
NLQGENEEEDSGAIVTVK	966.4632	2	1390.669	1	y13
NLQGENEEEDSGAIVTVK	966.4632	2	1276.626	1	y12
NLQGENEEEDSGAIVTVK	966.4632	2	1147.584	1	y11
NLQGENEEEDSGAIVTVK	966.4632	2	1018.541	1	y10
NLQGENEEEDSGAIVTVK	966.4632	2	889.4984	1	y9
NLQGENEEEDSGAIVTVK[HeavyK]	970.4703	2	1398.683	1	y13
NLQGENEEEDSGAIVTVK[HeavyK]	970.4703	2	1284.64	1	y12
NLQGENEEEDSGAIVTVK[HeavyK]	970.4703	2	1155.598	1	y11
NLQGENEEEDSGAIVTVK[HeavyK]	970.4703	2	1026.555	1	y10
NLQGENEEEDSGAIVTVK[HeavyK]	970.4703	2	897.5126	1	y9
FIAEGHPLSLK	404.5642	3	460.3124	1	y4
FIAEGHPLSLK	404.5642	3	557.3652	1	y5
FIAEGHPLSLK	404.5642	3	694.4241	1	y6
FIAEGHPLSLK	404.5642	3	751.4456	1	y7
FIAEGHPLSLK	404.5642	3	880.4882	1	y8
FIAEGHPLSLK	404.5642	3	951.5253	1	y9
FIAEGHPLSLK[HeavyK]	407.2356	3	468.3266	1	y4
FIAEGHPLSLK[HeavyK]	407.2356	3	565.3794	1	y5
FIAEGHPLSLK[HeavyK]	407.2356	3	702.4383	1	y6
FIAEGHPLSLK[HeavyK]	407.2356	3	759.4598	1	y7
FIAEGHPLSLK[HeavyK]	407.2356	3	888.5023	1	y8
FIAEGHPLSLK[HeavyK]	407.2356	3	959.5394	1	y9
VESEGGLIQTWNSQHPELK	718.0271	3	854.444	2	y15
VESEGGLIQTWNSQHPELK	718.0271	3	962.4813	2	y17
VESEGGLIQTWNSQHPELK	718.0271	3	486.2917	1	y4
VESEGGLIQTWNSQHPELK	718.0271	3	1367.67	1	y11
VESEGGLIQTWNSQHPELK	718.0271	3	1239.611	1	y10
VESEGGLIQTWNSQHPELK	718.0271	3	1138.563	1	y9
VESEGGLIQTWNSQHPELK	718.0271	3	740.8805	2	y12
VESEGGLIQTWNSQHPELK	718.0271	3	445.1924	1	b3
VESEGGLIQTWNSQHPELK[HeavyK]	720.6985	3	858.4511	2	y15
VESEGGLIQTWNSQHPELK[HeavyK]	720.6985	3	966.4884	2	y17
VESEGGLIQTWNSQHPELK[HeavyK]	720.6985	3	494.3059	1	y4
VESEGGLIQTWNSQHPELK[HeavyK]	720.6985	3	1375.684	1	y11
VESEGGLIQTWNSQHPELK[HeavyK]	720.6985	3	1247.625	1	y10
VESEGGLIQTWNSQHPELK[HeavyK]	720.6985	3	1146.578	1	y9
VESEGGLIQTWNSQHPELK[HeavyK]	720.6985	3	744.8876	2	y12
VESEGGLIQTWNSQHPELK[HeavyK]	720.6985	3	445.1924	1	b3
LITLAIPVNKPGR	464.6292	3	457.2876	1	y4
LITLAIPVNKPGR	464.6292	3	571.3305	1	y5
LITLAIPVNKPGR	464.6292	3	670.3989	1	y6
LITLAIPVNKPGR	464.6292	3	767.4517	1	y7
LITLAIPVNKPGR	464.6292	3	880.5358	1	y8
LITLAIPVNKPGR	464.6292	3	951.5729	1	y9
LITLAIPVNKPGR	464.6292	3	1064.657	1	y10
LITLAIPVNKPGR	464.6292	3	1165.705	1	y11
LITLAIPVNKPGR[HeavyR]	467.9653	3	467.2959	1	y4
LITLAIPVNKPGR[HeavyR]	467.9653	3	581.3388	1	y5
LITLAIPVNKPGR[HeavyR]	467.9653	3	680.4072	1	y6
LITLAIPVNKPGR[HeavyR]	467.9653	3	777.46	1	y7
LITLAIPVNKPGR[HeavyR]	467.9653	3	890.544	1	y8
LITLAIPVNKPGR[HeavyR]	467.9653	3	961.5811	1	y9
LITLAIPVNKPGR[HeavyR]	467.9653	3	1074.665	1	y10
LITLAIPVNKPGR[HeavyR]	467.9653	3	1175.713	1	y11
FYLAGNQEQEFLQYQPQK	1116.042	2	663.3455	1	y5
FYLAGNQEQEFLQYQPQK	1116.042	2	791.4041	1	y6
FYLAGNQEQEFLQYQPQK	1116.042	2	904.4882	1	y7
FYLAGNQEQEFLQYQPQK	1116.042	2	1051.557	1	y8
FYLAGNQEQEFLQYQPQK	1116.042	2	1180.599	1	y9
FYLAGNQEQEFLQYQPQK	1116.042	2	372.2236	1	y3
FYLAGNQEQEFLQYQPQK[HeavyK]	1120.0491	2	671.3597	1	y5
FYLAGNQEQEFLQYQPQK[HeavyK]	1120.0491	2	799.4183	1	y6
FYLAGNQEQEFLQYQPQK[HeavyK]	1120.0491	2	912.5023	1	y7
FYLAGNQEQEFLQYQPQK[HeavyK]	1120.0491	2	1059.571	1	y8
FYLAGNQEQEFLQYQPQK[HeavyK]	1120.0491	2	1188.613	1	y9
FYLAGNQEQEFLQYQPQK[HeavyK]	1120.0491	2	380.2378	1	y3
VLIVPQNFVVAAR	713.4324	2	425.3117	1	b3
VLIVPQNFVVAAR	713.4324	2	501.2797	2	y9
VLIVPQNFVVAAR	713.4324	2	1001.552	1	y9
VLIVPQNFVVAAR	713.4324	2	326.2433	1	b2
VLIVPQNFVVAAR	713.4324	2	904.4994	1	y8
VLIVPQNFVVAAR	713.4324	2	1100.621	1	y10
VLIVPQNFVVAAR	713.4324	2	776.4408	1	y7
VLIVPQNFVVAAR[HeavyR]	718.4365	2	425.3117	1	b3
VLIVPQNFVVAAR[HeavyR]	718.4365	2	506.2838	2	y9
VLIVPQNFVVAAR[HeavyR]	718.4365	2	1011.56	1	y9
VLIVPQNFVVAAR[HeavyR]	718.4365	2	326.2433	1	b2
VLIVPQNFVVAAR[HeavyR]	718.4365	2	914.5076	1	y8
VLIVPQNFVVAAR[HeavyR]	718.4365	2	1110.629	1	y10
VLIVPQNFVVAAR[HeavyR]	718.4365	2	786.4491	1	y7
DTVDGWFNIER	676.3174	2	531.288	1	y4
DTVDGWFNIER	676.3174	2	921.4572	1	y7
DTVDGWFNIER	676.3174	2	678.3564	1	y5
DTVDGWFNIER	676.3174	2	1036.484	1	y8
DTVDGWFNIER	676.3174	2	864.4357	1	y6
DTVDGWFNIER[HeavyR]	681.3216	2	541.2963	1	y4
DTVDGWFNIER[HeavyR]	681.3216	2	931.4655	1	y7
DTVDGWFNIER[HeavyR]	681.3216	2	688.3647	1	y5
DTVDGWFNIER[HeavyR]	681.3216	2	1046.492	1	y8
DTVDGWFNIER[HeavyR]	681.3216	2	874.444	1	y6
DGPLEFFGFSTSAR	765.8648	2	1148.536	1	y10
DGPLEFFGFSTSAR	765.8648	2	1019.494	1	y9
DGPLEFFGFSTSAR	765.8648	2	872.4255	1	y8
DGPLEFFGFSTSAR	765.8648	2	725.3571	1	y7
DGPLEFFGFSTSAR	765.8648	2	1415.695	1	y13
DGPLEFFGFSTSAR	765.8648	2	1358.673	1	y12
DGPLEFFGFSTSAR	765.8648	2	1261.621	1	y11
DGPLEFFGFSTSAR[HeavyR]	770.8689	2	1158.545	1	y10
DGPLEFFGFSTSAR[HeavyR]	770.8689	2	1029.502	1	y9
DGPLEFFGFSTSAR[HeavyR]	770.8689	2	882.4338	1	y8
DGPLEFFGFSTSAR[HeavyR]	770.8689	2	735.3654	1	y7
DGPLEFFGFSTSAR[HeavyR]	770.8689	2	1425.703	1	y13
DGPLEFFGFSTSAR[HeavyR]	770.8689	2	1368.682	1	y12
DGPLEFFGFSTSAR[HeavyR]	770.8689	2	1271.629	1	y11

*Soy allergen MRM – Peptide sequence, precursor, and product masses and charge states along with product ion types for the multiplexed MRM allergen assay, are presented*.
